# Temperature-activity relationships in *Meligethes aeneus*: implications for pest management

**DOI:** 10.1002/ps.3860

**Published:** 2014-08-26

**Authors:** Andrew W Ferguson, Lucy M Nevard, Suzanne J Clark, Samantha M Cook

**Affiliations:** a Andrew Ferguson Science ConsultingLuton, Bedfordshire, UK; b AgroEcology Department, Rothamsted ResearchHarpenden, Hertfordshire, UK; c Computational and Systems Biology Department, Rothamsted ResearchHarpenden, Hertfordshire, UK

**Keywords:** flight threshold, feeding, oviposition, *Brassica napus*, decision support, risk assessment

## Abstract

**BACKGROUND:**

Pollen beetle (*Meligethes aeneus* F.) management in oilseed rape (*Brassica napus* L.) has become an urgent issue in the light of insecticide resistance. Risk prediction advice has relied upon flight temperature thresholds, while risk assessment uses simple economic thresholds. However, there is variation in the reported temperature of migration, and economic thresholds vary widely across Europe, probably owing to climatic factors interacting with beetle activity and plant compensation for damage. The effect of temperature on flight, feeding and oviposition activity of *M. aeneus* was examined in controlled conditions.

**RESULTS:**

Escape from a release vial was taken as evidence of flight and was supported by video observations. The propensity to fly followed a sigmoid temperature–response curve between 6 and 23 °C; the 10, 25 and 50% flight temperature thresholds were 12.0–12.5 °C, 13.6–14.2 °C and 15.5–16.2 °C, respectively. Thresholds were slightly higher in the second of two flight bioassays, suggesting an effect of beetle age. Strong positive relationships were found between temperature (6–20 °C) and the rates of feeding and oviposition on flower buds of oilseed rape.

**CONCLUSION:**

These temperature relationships could be used to improve *M. aeneus* migration risk assessment, refine weather-based decision support systems and modulate damage thresholds according to rates of bud damage. © 2014 Society of Chemical Industry

## INTRODUCTION

Pollen beetles (*Meligethes aeneus* F.) are a major target of spring-applied insecticides in oilseed rape (OSR) (*Brassica napus* L.) in Europe.^1^–^3^ They cause feeding damage to flower buds, resulting in bud abscission and loss of yield.^4^–^7^ Improving the sustainability of pollen beetle management has become an increasingly urgent issue in the light of evidence for overuse of insecticides in their control and the threat posed by widespread insecticide resistance.^8^–^13^ There is a need for detailed studies on pollen beetle biology and behaviour to underpin the development of robust and practical decision support tools to improve targeting of control methods and stewardship of insecticides.^1^

Temperature strongly influences the activity and behaviour of pests and their interaction with host plants, and it is a key input to many decision support systems (DSSs) for pest management.^8^,^14^–^18^ DSSs generally address two different processes, risk prediction and risk assessment, providing guidance firstly as to when a crop is likely to be at risk and secondly on how the severity of risk can be assessed (and hence whether control is needed). Until recently, risk prediction advice for pollen beetles in Europe has relied upon the use of simple flight temperature thresholds. For example, current UK advice states that adult pollen beetles migrate at temperatures over 15 °C, and that oilseed rape crops are at risk from pollen beetle damage when they are at the ‘green-yellow bud stage’ (BBCH growth stage 51–59).^19^,^20^ This flight threshold accords with the generally accepted temperature threshold for mass migration of pollen beetles in Europe.^21^–^25^

A simple temperature threshold is unlikely to define good migration conditions sufficiently for it to be a wholly reliable risk prediction tool for pollen beetles. Current knowledge of flight–temperature relationships are derived from field studies, and flight has been reported at temperatures significantly below 15 °C.^26^–^29^ For example, Ferguson *et al.*^29^ found pollen beetle flight within a plot of OSR at 12 °C, while Láska and Kocourek^27^ reported flight at 10.2 °C and mass flight at 12.3 °C. A more sophisticated understanding of pollen beetle flight–temperature relationships is needed. Some authors assert the importance of accumulated periods of temperatures above a lower threshold for enabling mass flight.^28^,^30^ Recently the DSS ‘proPlant expert’ (http://www.proplantexpert.com) has become widely used throughout Europe for pest management in OSR crops.^31^–^34^ This web-based DSS uses phenological models driven by weather data (air temperature, rainfall, sunshine and wind speed) automatically downloaded from local meteorological stations. It provides local three-day forecasts of pollen beetle migration risk and indicates days when crop monitoring is needed. An analysis of the relationship between pollen beetle flight and temperature under controlled laboratory conditions could provide valuable data for refining such models.

Once a potential risk has been identified using the forecasting element of the DSS, the level of risk must be assessed, usually by comparing the abundance of the pest with an established economic threshold. This implies the existence of a simple relationship between pest numbers and yield loss, yet recommended pollen beetle control thresholds vary markedly between European countries.^2^ This suggests that local conditions may influence the ability of plants to withstand injury. It has been proposed that more robust economic thresholds could be developed if they were moderated by field-specific information such as crop cultivar, crop density, crop vigour and soil conditions, as well as pest numbers.^1^,^35^,^36^ However, there is a potential conflict between the need for accuracy of risk assessment, the need to inspire confidence in the DSS and the ease of its employment. There is good evidence that many growers do not use existing pollen beetle control thresholds because, as with many decision support tools, they are perceived to be onerous, the labour costs of employing them being significant in the context of the low cost of control chemicals and the potential to tank-mix them with fungicide applications.^8^,^9^,^36^

There may be potential to use weather data to improve risk assessment without the need for extra sampling input by the user. The impact of pollen beetles on the yield of a plant is likely to depend not only on their abundance but also on the balance between pollen beetle activity, the rate of plant development and the ability of the plant to compensate for damage.^17^,^37^–^39^ These processes are probably differentially influenced by temperature. The present authors propose that model-based DSSs could be extended to model the weather-dependent relationship between pest numbers monitored on the crop and damage in WOSR. This could enable the DSS not only to forecast immigration risk but also the degree of damage risk associated with any sampled abundance of beetles in the context of expected weather.

The objective of this study is better characterisation of the influence of temperature on pollen beetle activity. Using a novel bioassay conducted under controlled laboratory conditions, an examination is made of evidence for a flight temperature threshold in pollen beetles, and for the first time the influence of temperature on feeding and oviposition rates on oilseed rape buds is characterised.

## MATERIALS AND METHODS

### Pollen beetles

Overwintered-generation pollen beetles were collected by sweep net from a crop of flowering winter OSR on Rothamsted Farm, Hertfordshire, United Kingdom, between 21 May and 5 June 2012. Emergence traps set out on the crop indicated that new-generation pollen beetles had not emerged by 5 June. The beetles collected were maintained until use in groups of up to 500 in ventilated plastic boxes (174 × 116 × 60 mm) with cut flowering racemes of glasshouse-grown spring OSR (cv. Heros) at 8 °C with a 16/8 h L/D cycle. All experiments and observations took place in June and July 2012.

### Controlled environment conditions

All experiments and observations were made in insect cages in four matched controlled environment (CE) cabinets with opaque white walls (Conviron Adaptis A1000, http://www.conviron.com). Constant light levels were maintained throughout, the insect cages receiving 260–270 µmol m^−2^ s^−1^ of PAR light (400–700 nm). Temperatures were set to achieve the desired temperature treatments (6, 8, 9, 10, 11, 12, 13, 14, 16, 18, 20 or 23 °C) within flight bioassay cages. Temperatures in all cages were recorded at 1 min intervals throughout experiments using type K thermocouple sensors and Testo 177-T4 temperature dataloggers (http://www.testo.co.uk). This allowed all analyses to be related to the actual temperatures experienced by the pollen beetles.

### Relationship between temperature and flight

#### Flight bioassay

**Figure 1:**
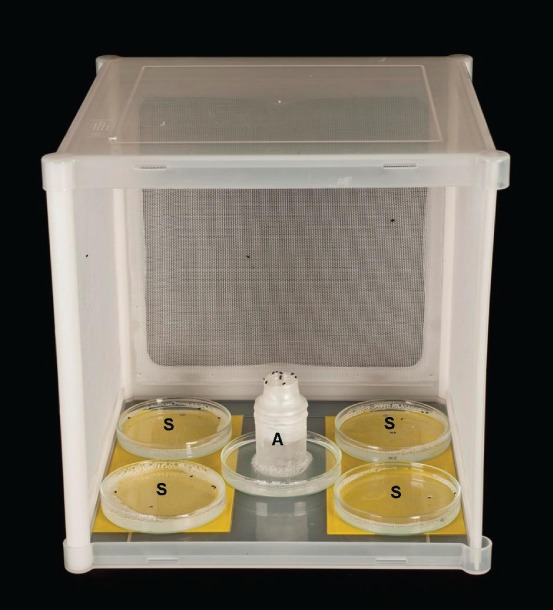
Insect flight cage (‘Bugdorm-1’, http://www.megaview.com.tw) with the front removed to demonstrate the experimental set-up for the flight bioassay. (A) The central release point, consisting of the release vial containing ca 50 pollen beetles at the start of the run, the ice jacket around the release vial and the central water trap to prevent beetle escape by walking. (S) Four ‘sink’ water traps to reduce the chance that pollen beetles that escaped the central release point would return to the central water trap.

In preparation for each test, 50 pollen beetles of mixed sex were placed into a glass ‘release vial’, 73 × 22 mm internal diameter, enclosed with a screw cap and chilled to 0 °C to render them inactive. Immediately prior to its introduction to the flight cage, the release vial was packed in ice inside an open-topped plastic vial, 60 × 37 mm internal diameter, forming an ice jacket. The top of the release vial protruded ca 20 mm above the ice jacket, to which it was sealed using Parafilm® M sealing film, to prevent beetles from falling into the ice during the experiment (Fig. 1).

Each test was conducted in a well-ventilated translucent polypropylene insect flight cage (300 × 300 × 300 mm; ‘Bugdorm-1’, http://www.megaview.com.tw) on an open shelf in the middle of one of the four CE cabinets. The temperature probe (see above) was positioned in the middle of the cage. On the floor of each insect cage there were five water traps, each consisting of a glass petri dish base (95 mm diameter) filled with a 2.5% solution of detergent (Teepol) in tap water. One trap was placed in each corner of the cage, set against a yellow background to improve its attractiveness to pollen beetles, and the fifth was placed in the centre (Fig. 1).

On introduction into the flight cage, the release vial and ice jacket were placed in the middle of the central water trap, and the screw cap of the release vial was removed to allow beetles to exit. The release vial, ice jacket and central water trap will hereafter be referred to as the ‘release point’. As the ice melted, the vial warmed, so that the beetles experienced the temperature set within the cage and their activity level responded accordingly. The purpose of the central water trap was to prevent escape of beetles from the release point except by flight. The other traps served as sinks for those that had escaped the release point, to reduce the chance of their return to the release point. At 20 h after the introduction of beetles into the cage, the number of beetles that had escaped the release point was counted and was taken to be evidence of the propensity to fly at the treatment temperature.

Tests were run in two experiments from 4 to 19 June and from 9 to 24 July 2012, respectively. In the first experiment, eight temperatures, in increments of 2 °C from 6 to 20 °C, were tested in four CE cabinets (one flight cage per cabinet), using a balanced incomplete block design. Four temperatures were simultaneously tested in each of 14 twenty-hour runs (incomplete blocks), and each temperature was replicated 7 times. Following a preliminary examination of the data from the first experiment, additional test temperatures were chosen for a second flight experiment to increase confidence in the temperature–response model. To enrich data in the temperature range critical to spring immigration, temperatures of 9, 11 and 13 °C were chosen. To these were added 23 °C, to extend the range of temperatures upwards, and two temperatures already tested, 12 and 18 °C, to link the two datasets. These six temperatures were tested in three CE cabinets, again using a balanced incomplete block design, three temperatures being tested in each of ten runs and each temperature being replicated 5 times.

#### Video observations

To provide evidence that the escape of pollen beetles from the release point in the flight bioassay was indeed indicative of flight, video observations were undertaken at each of the temperatures used in the bioassay, using the same experimental cage and pollen beetle release procedures (Fig. 1). One temperature was tested at a time in a single CE cabinet. At each temperature, the behaviour of one set of 50 beetles was closely observed by video for 7 h. The camera (Sony DCR-SX33E ‘Handycam’) was enclosed within the cabinet, its lens protruding through the entrance sleeve to the flight cage and focused on the top of the release vial, giving an in-focus field of view of ca 45 × 80 mm wide. Recordings were made at 25 frames s^−1^. For each temperature, the video footage was observed to record the method of movement used by each beetle as it left the field of view on escape from the release vial. Each beetle escape was allocated to one of two movement categories: ‘closed wings’ or ‘open wings’. The closed wing category included beetles that walked from the top of the release vial down the outside of the vial and out of view (see supplementary material videoclip S1 http://youtu.be/m1Jjs2WzLVc), as well as those that dropped from view without opening their elytra (supplementary material videoclip S2 http://youtu.be/3-jdVCk9woo). The open wing category included any beetle that departed the release vial in the air, with its elytra open and its wings spread (supplementary material videoclips S3 http://youtu.be/bb-mprRI7xA and S4 http://youtu.be/8eGbYQzAHJ4, showing upward and downward trajectory flights, respectively), and this was taken as evidence of a propensity to fly. Any beetles that could not be seen well enough to determine whether their elytra were open were excluded from observations. This was usually because they were out of the narrow plane of focus or were obscured by other beetles when they made their movement. Temperatures were tested in randomised order, and all temperatures were observed once during the experiment. All video observations were made from 12 to 31 July 2012.

### Relationship between temperature and bud damage: feeding and oviposition bioassay

The influence of temperature on the feeding and oviposition of female pollen beetles on buds was tested in a replicated bioassay. Gravid female beetles were selected for use in the bioassay using a protocol similar to that of Hopkins and Ekbom:^40^ 20 unsexed beetles were enclosed singly at room conditions for a minimum of 12 h (including overnight) on cut racemes of glasshouse-grown spring OSR with five or more flower buds of optimum size for oviposition (2–3 mm long).^5^,^40^,^41^ The buds were examined for oviposition damage, and the four beetles that had oviposited into the most buds (minimum two buds) were selected for use in the feeding and oviposition bioassay the following day.

Tests were conducted in small cylindrical cages, 45 × 32 mm inside diameter, concurrently with experiment 1 of the flight bioassay (4–19 June 2012). One small cylindrical cage was placed alongside the flight cage in each of the four CE cabinets used for the flight bioassay (see above). Each small cage was made from a 60 mL transparent polystyrene vial (Sterilin Ltd, Newport, Gwent, UK), with the bottom removed and replaced with a plug of Oasis® Floral Foam (Smithers-Oasis UK Ltd, Washington, Tyne and Wear, UK) that was fully hydrated and wrapped in plastic film. The temperature probe (see above) was inserted through the top of the cage which was closed with a polyethylene screw cap into which a ventilation hole (30 mm diameter) was cut and covered in nylon mesh. Each cage contained a cut raceme of glasshouse-grown spring OSR (cv. Heros) with its stem inserted into the floral foam and onto which a single female beetle was introduced. Racemes presented were selected to be as uniform as possible, with a minimum of ten buds of optimum size for oviposition (2–3 mm long), together with the terminal rosette of smaller developing flower buds, but no larger buds. This provided females with opportunities for oviposition and feeding well in excess of daily bud usage reported by other authors (under glasshouse conditions: 1.2 buds per female for oviposition and 1.7 buds for feeding;^5^ in the field: ca three buds per female for oviposition^41^). After 20 h at the test temperature in the CE cabinet, the beetle was removed from the cage and all buds 0.5 mm or longer were examined to count the number with feeding damage, the number with eggs and the total number of eggs laid. Preliminary observations indicated that temperatures in the small cages were about 0.5 °C higher than in the flight cages. Thus, the temperatures recorded in the small cages during the feeding and oviposition bioassay ranged from ca 6.5 °C to ca 20.5 °C, in increments of ca 2 °C.

Owing to a temperature recording failure, replication at target temperatures 12, 14, 16 and 20 °C in experiment 1 of the flight bioassay and replication in the bud damage bioassay were reduced from seven to six cages per target temperature.

### Statistical analysis

#### Flight bioassay and video observations

The numbers (out of ca 50) of beetles escaping per cage in the flight bioassay were regressed on the maximum temperature per cage during the experiment (logged base 10) using non-linear logistic regression (GLM with binomial error and logit link). Data from the two flight bioassay experiments were analysed together. A series of nested models maximally allowing ‘immunity’ (i.e. allowing for a proportion of beetles that would not fly at any temperature), and allowing all intercept and slope parameters to differ between experiments, were fitted and compared; from these, the best-fit parsimonious model was chosen. For the video observations, the binary response (wings open or closed) of each insect was analysed by logistic regression in relation to the temperature recorded in the flight cage at the time of escape (logged base 10). For both the flight bioassay and the video observations, the temperatures at which 10, 25 or 50% of beetles (allowing for immunity) showed evidence of a propensity to fly (‘flight temperature thresholds’ denoted by FT_10_, FT_25_ and FT_50_, respectively) were estimated from the chosen model.

#### Feeding and oviposition bioassay

Counts from each cage of the bud damage bioassay were analysed using linear mixed models and restricted maximum likelihood to describe their relationship with the mean temperatures recorded within the cages during each run. The data were analysed either as untransformed counts or, if necessary, as counts transformed to a natural log scale [ln (*y* + 1)] to stabilise the variance. Straight-line regression relationships were fitted (fixed model), and the blocking structure was accounted for within the random model. All analyses were performed using Genstat (http://www.vsni.co.uk/).

## RESULTS

### Temperatures in cages

For both bioassays and in the video observations, temperature series with well-spaced intervals were achieved in cages (Table 1). However, in each case, temperatures departed somewhat from the target temperatures, and so actual recorded temperatures were used for analyses. In the flight bioassays, mean cage temperatures were within 0.2 °C of the target, and mean maxima were within 0.3 °C of the mean (Table 1). In the video observations, the mean temperature at the time of beetles' escape from the release point was usually slightly higher than the target temperature (Table 1). This probably, at least in part, reflects a tendency to move more at warmer times in the thermostatically controlled cycling of temperature around the set point. Consistent with this, in preliminary analysis of the flight temperature bioassay, a model using the maximum cage temperature gave a slightly better fit to the data than one using the mean cage temperature, and therefore maximum temperature was used for the full analysis. Mean temperatures in the cages of the bud damage bioassay were 0.4–0.7 °C higher than in cages of the flight bioassay run at the same time (Table 1).

**Table 1:** Mean temperatures recorded in flight bioassay cages, video observations and bud damage bioassay. Standard errors of means (SEM) are given in parentheses

	Flight bioassay				Video observations		Bud damage bioassay	
			
		Temperatures per cage (°C)						
							
Target temperature (°C)	Experiment number	Mean	Mean maximum	Number of cages	Mean escape temperature per beetle (°C)	Number of beetles	Mean temperature per cage (°C)	Number of cages
6	1	6.2 (0.05)	6.5 (0.04)	7	5.9 (0.03)	24	6.7 (0.09)	7
8	1	8.2 (0.06)	8.4 (0.07)	7	8.4 (0.04)	20	8.6 (0.13)	7
9	2	9.2 (0.09)	9.5 (0.09)	5	9.7 (0.05)	40		
10	1	10.0 (0.07)	10.2 (0.07)	7	10.5 (0.04)	41	10.6 (0.09)	7
11	2	10.9 (0.11)	11.2 (0.13)	5	11.3 (0.02)	42		
12	1	12.2 (0.06)	12.5 (0.06)	6	12.4 (0.02)	48	12.7 (0.11)	6
12	2	12.1 (0.11)	12.3 (0.12)	5				
13	2	12.9 (0.14)	13.2 (0.12)	5	13.2 (0.02)	46		
14	1	14.2 (0.07)	14.5 (0.08)	6	14.4 (0.02)	46	14.7 (0.13)	6
16	1	16.1 (0.09)	16.4 (0.11)	6	16.0 (0.04)	48	16.7 (0.14)	6
18	1	18.2 (0.04)	18.4 (0.04)	7	18.1 (0.03)	40	18.7 (0.07)	7
18	2	18.0 (0.11)	18.3 (0.07)	5				
20	1	19.9 (0.05)	20.2 (0.05)	6	20.2 (0.02)	46	20.4 (0.11)	6
23	2	23.0 (0.08)	23.3 (0.06)	5	23.3 (0.02)	45		

### Relationship between temperature and flight

#### Flight bioassay

**Figure 2:**
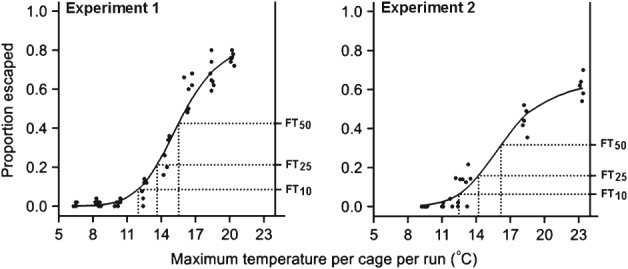
Fitted temperature–response models for flight bioassay experiments 1 and 2, indicating estimated flight threshold values FT_10_ [experiment 1, 12.0 °C, 95% confidence limits (CL) 11.5, 12.4; experiment 2, 12.5 °C, 95% CL 11.8, 13.2), FT_25_ (experiment 1, 13.6 °C, 95% CL 13.1, 14.2; experiment 2, 14.2 °C, 95% CL 13.4, 15.1) and FT_50_ (experiment 1, 15.5 °C, 95% CL 14.8, 16.4; experiment 2, 16.2 °C, 95% CL 15.2, 17.5). All axes and FT estimates are back-transformed from the log_10_ scale to the natural scale. Escape from the release point was taken as evidence of a propensity to fly. Filled circles represent the observed data, i.e. the proportion of pollen beetles in each cage that escaped the release point, plotted against the maximum temperature recorded in the cage during the run (*N* = 56 runs for experiment 1 and 30 runs for experiment 2).

The relationship between the proportion of beetles escaping and the temperature in the two experiments was best described by separate lines for each experiment, but with a common slope (Fig. 2). The chosen model for the data included five terms: two separate ‘immunity’ parameters (i.e. estimates of the proportion of beetles that would not escape whatever the temperature), two *y*-axis intercepts (logit proportion of beetles) and a single regression coefficient common to both experiments (slope) (Table 2). In experiment 1, 10% of ‘non-immune’ beetles showed evidence of a propensity to fly at 12 °C (FT_10_), rising to 50% at 15.5 °C (FT_50_) (Fig. 2). In experiment 2, started 35 days later than experiment 1, values of FT_10_, FT_25_ and FT_50_ were found to be about half a degree higher than in experiment 1, although their 95% confidence limits overlapped with the values from experiment 1 (Fig. 2). The model also suggested that in experiment 2 about 37% of beetles were ‘immune’, i.e. would not escape at any temperature, compared with 15% in experiment 1 (Table 2).

**Table 2:** Parameter estimates from the final non-linear logistic regression model fitted to the proportion of pollen beetles escaping from the release point in the flight bioassay and the proportion of beetles opening their wings in the video observations. SEM given in parentheses

	Flight bioassay		
		
	Experiment 1	Experiment 2	Video observations
Proportion of beetles ‘immune’	0.15 (0.052)	0.37 (0.047)	0.20 (0.046)
Intercept (logit proportion of beetles)	−23.2 (1.82)	−23.5 (1.83)	−29.2 (5.63)
Slope (log_10_ temperature)	19.5 (1.64)	19.5 (1.64)	26.1 (5.15)

#### Video observations

**Figure 3:**
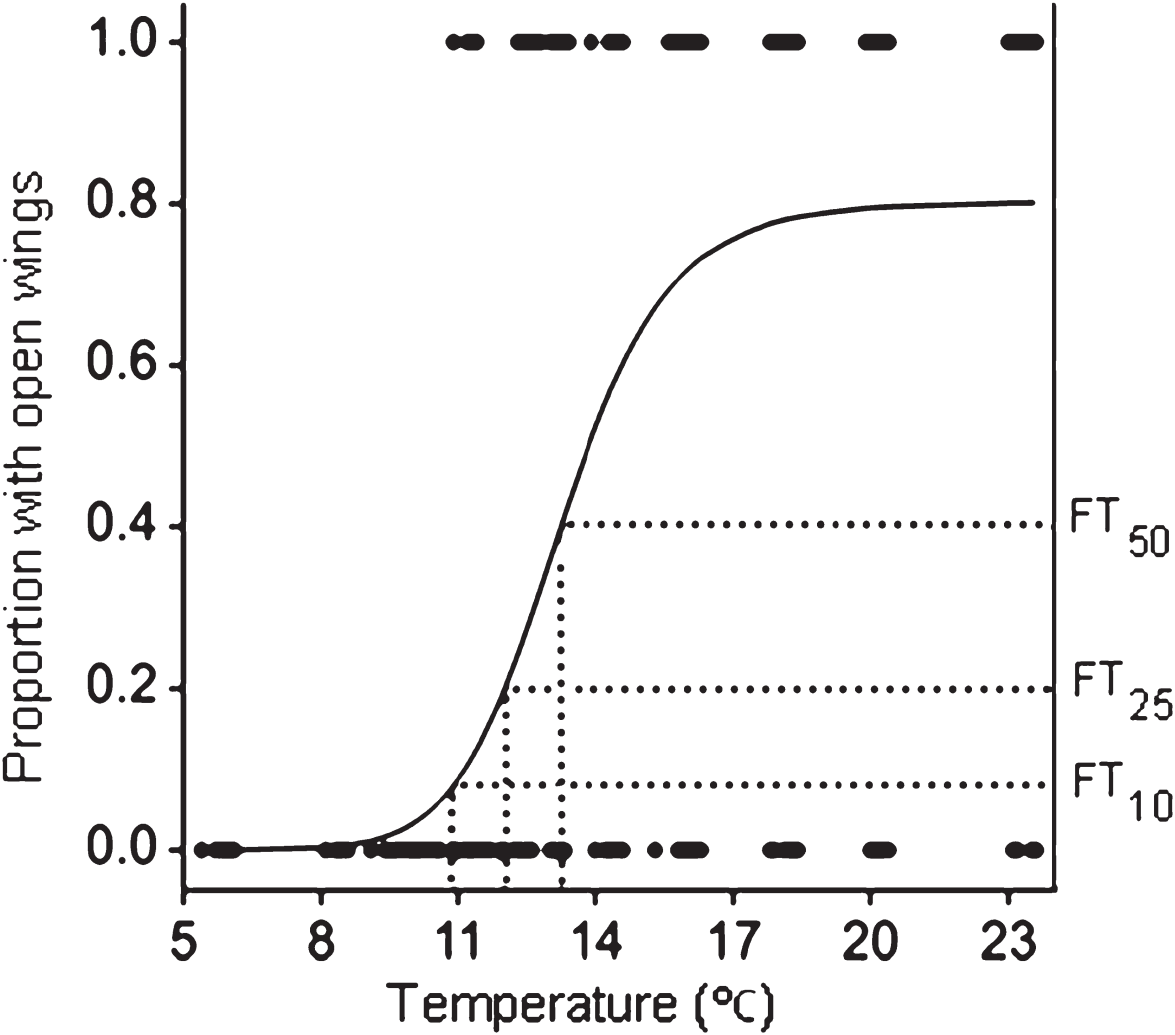
Fitted temperature–response model for video observations of flight activity, indicating estimated pollen beetle flight threshold values FT_10_ (10.9 °C, 95% CL 9.9, 11.5), FT_25_ (12.0 °C, 95% CL 11.4, 12.6) and FT_50_ (13.2 °C, 95% CL 12.7, 14.2). Both axes and all FT estimates are back-transformed from the log_10_ scale to the natural scale. Wing opening is taken to be indicative of a propensity to fly. Filled circles represent the observed data for each of the *N* = 486 pollen beetles that escaped the release point, plotted against the cage temperature recorded at the moment of escape. For each beetle, the observed data are of binary form, representing the wing status (open or closed).

The fitted temperature–response model for the proportion of beetles opening their wings in video observations showed a similar sigmoidal form to that from the flight bioassay (Fig. 3, Table 2). Estimates of the three flight temperature threshold values FT_10_, FT_25_ and FT_50_, based on video observations, were 1.6–2.0 °C lower than in experiment 2 of the flight bioassay, which was conducted concurrently, and there was a lower level of ‘immunity’ (Figs 2 and 3, Table 2).

### Relationship between temperature and bud damage

**Figure 4:**
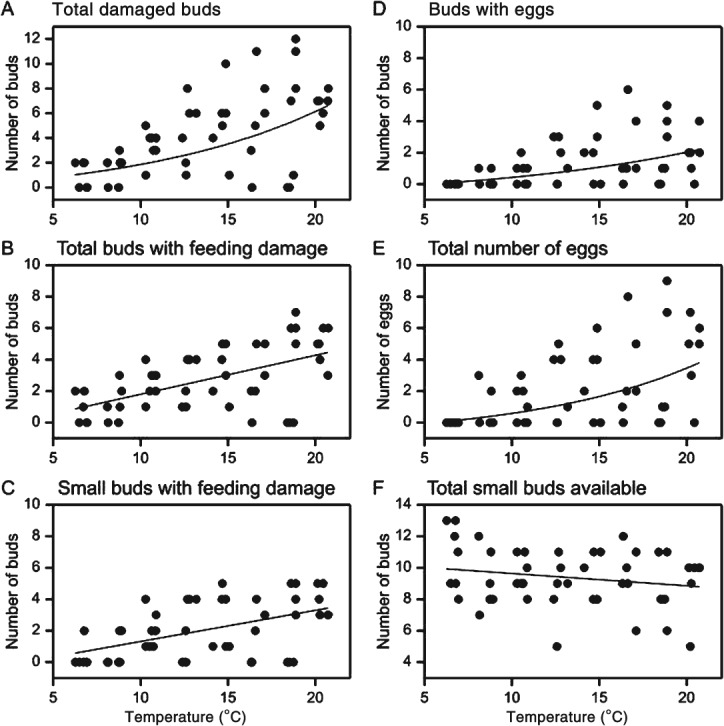
Relationships of temperature with pollen beetle bud damage and egg laying on oilseed rape racemes. Regression lines are fitted by linear mixed model analysis. Curves in (A), (D) and (E) represent values back-transformed from the natural log scale. (A) Total number of damaged buds of all sizes (slope 0.42, SE 0.070; intercept −1.64, SE 1.073; *F*_1,41.7_ = 36.8, *P* < 0.001). (B) Total number of buds of all sizes with feeding damage (slope 0.25, SE 0.047; intercept −0.68, SE 0.698; *F*_1,42.9_ = 27.8, *P* < 0.001). (C) Number of small buds (length ≥ 0.5 mm, < 2 mm) with feeding damage (slope 0.20, SE 0.046; intercept −0.66, SE 0.660; *F*_1,50.0_ = 18.1; *P* < 0.001). (D) Number of buds with eggs (slope 0.17, SE 0.037; intercept −1.03, SE 0.564; *F*_1,41.8_ = 21.3, *P* < 0.001). (E) Total number of eggs laid (slope 0.33, SE 0.052; intercept −2.23, SE 0.846; *F*_1,40.5_ = 38.6, *P* < 0.001). (F) Total number of small buds available (slope −0.08, SE 0.054; intercept 10.42, SE 0.773; *F*_1,45.6_ = 2.1, *P* = 0.16, no significant relationship).

Both the numbers of buds damaged and the numbers of eggs laid by beetles showed strong positive relationships with cage temperature (Fig. 4). The total number of damaged buds increased with temperature (Fig. 4A), the majority of buds being injured by feeding (Fig. 4B). Three-quarters of all buds that were fed upon were ‘small buds’ (length ≥ 0.5 mm, < 2 mm), increasing numbers of which were fed upon at higher temperatures (Fig. 4C). All eggs were laid into buds 2–3 mm long, and separate feeding damage lesions were not observed in buds with eggs. Buds with eggs contained a mean of 1.7 eggs, range 1–4. Both the number of buds with eggs and the total number of eggs laid increased with temperature (Figs 4D and E), the oviposition rate rising to about four eggs per beetle per 20 h at 20 °C (Fig. 4E). The maximum number of buds 2–3 mm long receiving any damage in any run was 7, and the maximum receiving eggs was 6, confirming that the minimum of ten buds in this size range that were offered to each beetle represented an excess to requirement. Similarly, the number of small buds available always exceeded the number damaged (mean 21% with feeding damage, range 0–80%). The total number of small buds presented, which had not been counted before each test, did not vary significantly with test temperature (Fig. 4F), suggesting that the temperature relationships in Figs 4A to E were not influenced by the number of small buds presented.

## DISCUSSION AND CONCLUSIONS

Strong behavioural responses to temperature in pollen beetle flight, feeding and egg-laying activity have been demonstrated and characterised. These temperature relationships have important implications for risk assessment and pest management.

### Relationship between temperature and flight

Flight bioassay data and video observations provided compelling evidence that the propensity to fly follows a sigmoid temperature–response curve in the 6–23 °C temperature range tested. When analysing samples of field populations of flying insects, it has become customary to allow for variable population size by plotting the data for each temperature as the proportion of samples in which flying insects were caught.^15^ This produces similar sigmoid temperature–response curves for many species.^16^,^42^–^44^ The assumption that beetle escapes in the flight bioassay were indicative of wing-powered movement was strongly supported by the similarity of the temperature response of wing opening in the video observations. However, firm evidence of the extent of powered flight proved elusive. In a preliminary analysis of the video data, the proportion of beetles that took an upward trajectory as they escaped the release point (e.g. supplementary material video clip S3 http://youtu.be/bb-mprRI7xA) was recorded as incontrovertible evidence of powered flight. This proportion only exceeded 50% at 20 and 23 °C, temperatures well above those where there is good evidence for mass migration in the field,^21^,^22^,^24^ suggesting that the tendency to engage in powered flight would be greatly underestimated by the number flying with an upward trajectory. Video observations using a camera with greater resolution and shutter speed and with a wider field of view would allow wing movement to be analysed directly.

The temperature range over which evidence was found for flight in pollen beetles under laboratory conditions agreed well with field observations. It was estimated that FT_10_, the 10% flight temperature threshold, fell in the range 10.9–12.5 °C, approximating reports in the literature of 10.2–13.5 °C for the lower limits of field-observed flight.^22^,^26^–^29^ Present estimates of 15.5 and 16.2 °C for FT_50_ in the flight temperature bioassay agree with advice that pollen beetle migration is likely at temperatures around 15 °C or above.^19^,^20^,^22^–^25^ This close match between field and laboratory observations of pollen beetle flight activity confirms that temperature (as distinct from factors that are correlated with temperature in the field, such as day length) is indeed a key factor limiting movement from overwintering sites to OSR crops in spring.^21^,^24^,^27^,^28^,^32^,^33^ The physiology of the pollen beetle may be adapted to limit energetically expensive flight activity until host plants at the preferred growth stage are available.

There was clear evidence that beetles showed less tendency to fly in the second of the two flight bioassays. The proportion of pollen beetles not expected to fly at any temperature was higher, and the flight temperature thresholds were also marginally higher. Although other factors cannot be ruled out, this is consistent with an effect of ageing, adults of this univoltine beetle having eclosed in July the previous year.^45^,^46^ It is important for modelling of migration risk that the slope of the relationship with temperature remained consistent between the two experiments and the intercepts were very close (Table 2). This suggests that the main difference between the experiments was in the size of the population of insects that would fly, and that the underlying relationship with temperature remained the same. This hypothesis should be tested by examining the flight–temperature response of beetles at different times through the year, both before and after overwintering, as their physiological condition changes with age. The responses of pollen beetles from the time of their emergence at overwintering sites until their migration to crops is of particular relevance to risk modelling for OSR.

Many authors have sought flight temperature thresholds as part of their studies of the influence of meteorological conditions on the migration of pest species.^14^–^16^,^47^ Now that sophisticated and near-real-time risk modelling is possible,^31^–^33^ it is appropriate to reassess the value of attempting to define flight temperature thresholds. A model of the relationship between temperature and migration is considerably more informative than an arbitrary threshold and could be interpreted in the light of the influence on migration risk of other meteorological variables and of population size.^9^ Nevertheless, the simple concept of the flight threshold is likely to remain useful to those planning pest monitoring and management, especially if without access to a DSS model, provided its limitations are understood.

### Relationship between temperature and bud damage

Strong positive relationships between temperature (6–20 °C) and the rates of pollen beetle feeding and oviposition on flower buds of oilseed rape have been demonstrated. More buds were damaged by feeding than by egg laying, consistent with other studies,^5^ and the relative importance of feeding damage is accentuated if males and non-egg-laying females (excluded from this study) are considered. This is noteworthy because adult feeding damage to a bud is usually more severe than that caused by larvae and is more likely to lead to bud abscission and yield loss.^45^ All eggs were laid in buds 2–3 mm long, as reported by other authors, whereas most feeding damage was in smaller buds, indicating a partial separation of resource allocation between the generations.^5^,^40^,^41^ The number of eggs laid per female (rising to an average of about 4 eggs per 20 h at 20 °C) was in keeping with other reported estimates ranging from 1.1–2.5 eggs female^−1^ day^−1^ in the glasshouse to 3.4–6.6 eggs female^−1^ day^−1^ in the field.^5^,^40^,^41^

### Implications for integrated pest management of pollen beetles

In this study, the first to examine pollen beetle flight–temperature relationships under controlled laboratory conditions, the authors have estimated parameters that could be used to refine the accuracy of weather-based phenological models that use local weather data to provide locally relevant forecasts of pollen beetle migration risk, such as ‘proPlant expert’.^31^–^33^ It is proposed that this analysis of the relationship between temperature and bud damage could help to extend a weather-based risk model to forecast not only the risk of pollen beetle migration into OSR but also the severity of damage risk when the beetles are on the crop.

Any damage risk model for pollen beetles in OSR should take into account the influence of weather both on beetle activity and on plant growth and compensation.^4^ Such a model has already been developed for the effect of pollen beetles on yield in spring OSR in Denmark.^17^ This model confirmed that, when growing conditions were better (more pre-immigration precipitation, higher post-immigration temperature), plants were better able to compensate for pollen beetle damage, and the negative impact on yield was reduced. Plant growth stage should also be a key input to damage risk models, as early beetle immigration is likely to result in continuing damage to buds over a longer period, whereas it is generally believed that damage becomes negligible once the crop is in flower.^20^,^45^ Ultimately, a risk model driven simply by automatically downloaded local weather data and weather forecasts and by plant growth stage could not only predict immigration risk but could also provide some information on the degree of damage risk and offer a control threshold moderated to local conditions.

Future work should seek to quantify the effects of weather variables and growth stage on the ability of OSR to compensate for pollen beetle damage and should expand on the present studies to include a cross-section of newly migrated individuals in spring, including males. For the foreseeable future, counts of pollen beetles on plants are likely to remain an essential input to damage risk models, and a key function of a good locally tailored DSS will be to enable sampling effort to be minimised and implemented at the appropriate time.
